# Heated tobacco products in Serbia: Tax differentials, relative prices, and excise tax pass-through

**DOI:** 10.18332/tid/231126

**Published:** 2026-10-05

**Authors:** Aleksandar Zdravković, Jovan Zubović, Boban Nedeljković

**Affiliations:** 1 Macroeconomics Department, Institute of Economic Sciences, Belgrade, Serbia; 2 Welfare Economics Department, Institute of Economic Sciences, Belgrade, Serbia

**Keywords:** heated tobacco products, tobacco taxation, tax pass-through, Serbia, tobacco control policy

## Abstract

**INTRODUCTION:**

Heated tobacco products (HTPs) have become a rapidly growing segment of the tobacco market in Serbia. This study examines the evolution of HTP prices, taxation, and relative market positioning in Serbia following their entry into the market.

**METHODS:**

A secondary data analysis was conducted using administrative and publicly available market data from Serbia. Monthly retail price and excise series covered Q3 2018 – Q1 2025, while aggregate tobacco sales data covered 2018–2023. Descriptive analysis was combined with first-difference panel regression models to estimate the pass-through effect, defined as the extent to which changes in HTP and cigarette excise taxes were reflected in HTP retail prices.

**RESULTS:**

HTPs accounted for approximately 2.5% of estimated tobacco packs sold in 2019 and 6.25% in 2023. While HTP prices moved closer to the medium cigarette segment, real HTP prices in 2023–2024 were lower than in 2018 and the excise burden on HTPs remained much lower than on cigarettes. The excise share of the leading HTP brand reached 13.4% in 2024, compared with 61.4% for the leading cigarette brand. The estimated direct pass-through coefficient for HTP excise changes was 0.26 (95% CI: -0.07–0.59). The point estimate was below full pass-through, but the confidence interval included zero. By contrast, cigarette excise changes were positively associated with HTP price changes (0.99; 95% CI: 0.61–1.36).

**CONCLUSIONS:**

In Serbia, HTP market expansion has taken place under a tax regime in which the effective tax burden on HTPs remains substantially lower than that on cigarettes. The estimated pass-through effects suggest that the industry may have absorbed part of recent HTP excise increases, although this finding should be interpreted cautiously given the observational design of the study and limited identifying variation. Overall, the results point to the relevance of considering closer alignment between HTP and cigarette taxation within tobacco tax policy.

## INTRODUCTION

Heated tobacco products (HTPs) have become one of the fastest-growing segments of the tobacco market in the last decade^1,2^. Their expansion has changed the strategic orientation of major tobacco companies and created new challenges for tobacco control and fiscal policy^1^. As cigarette markets have become more regulated and, in many countries, less dynamic, transnational tobacco companies have increasingly invested in non-combustible product lines, such as HTPs and electronic cigarettes^1^. At the same time, HTP use has grown across many countries. Global prevalence of ever use approached 5% by 2022, while in the European region lifetime use increased from just above 1% in 2016 to almost 7% by 2020^2^. This rapid uptake has been supported by intensive marketing, attractive device design, and repeated claims that HTPs are cleaner or less harmful alternatives to cigarettes^1,3^.

This development is important not only from the public health perspective, but also from the perspective of taxation and price regulation. The evidence on the health effects of HTPs remains incomplete and methodologically mixed. Some studies suggest lower exposure to certain harmful substances compared to continued cigarette smoking, but the available evidence is largely based on short-term studies and does not justify the conclusion that HTPs are harmless or without long-term risk^4-6^. Because of this, an important policy question is whether tax systems create incentives that make HTPs more attractive and more affordable than conventional tobacco products.

In many countries, excise systems were originally designed for manufactured cigarettes and were later adapted, often only partially, to novel tobacco products^7,8^. As a result, HTPs are frequently taxed under different rules and at lower effective levels than cigarettes^7,8^. Such differences can shape relative prices across product categories, affect affordability, encourage substitution, and create room for tax arbitrage and strategic pricing by the industry. When one product category is taxed more lightly than another, the expected public health and revenue effects of excise policy may be weakened, especially if tax increases are not fully reflected in retail prices.

Recent empirical evidence suggests that this issue is substantial in the case of HTPs. Studies indicate that demand for HTPs is more price-elastic than demand for cigarettes, which means that taxation may have a strong effect on their use^9,10^. Cross-price effects between cigarettes and HTPs further suggest that relative price changes may encourage switching between product categories. Another important issue is tax pass-through, that is, the extent to which excise tax changes are reflected in retail prices. Available evidence shows a contrast between cigarettes and HTPs. Cigarette excise increases are often fully shifted, or even over-shifted, to consumers, whereas HTP excise increases are more often under-shifted^7^. In such cases, producers absorb part of the tax increase instead of fully transferring it to final prices, which allows them to preserve or strategically adjust price differentials across products. Evidence from Montenegro points in the same direction, as large excise increases on HTPs were followed by much smaller increases in retail prices^11,12^. Recent evidence from Ukraine also documented price smoothing and differentiated pass-through across product categories during major tax reforms, illustrating how producers may adjust the timing and relative positioning of prices around scheduled tax changes^13^.

Serbia provides a relevant case for analyzing these issues. Although cigarettes remain the dominant tobacco product, HTPs have expanded rapidly since their market entry, as reflected in the marked growth of legal HTP sales and the number of active HTP brands between 2018 and 2023^14^. HTPs entered the Serbian market in 2018, and since then they have been taxed differently from manufactured cigarettes. While cigarettes are subject to both specific and ad valorem excise per pack, HTPs are taxed through a specific excise per kilogram of tobacco. In addition, Serbia introduced a phased schedule that links the HTP excise to the minimum excise on cigarettes by converting the cigarette-based minimum excise into an equivalent rate based on HTP tobacco content expressed per kilogram^15^. This institutional setting, together with the rapid market expansion, makes it possible to examine how a distinct excise design for HTPs affected their relative tax burden, price dynamics, and market positioning in relation to cigarettes.

The main aim of this study is to examine how HTP prices evolved relative to cigarette prices and how excise differentials translated into differences in tax burden. The analysis first describes the development of HTP sales, prices, and taxation after market entry. It then examines how HTP prices evolved relative to cigarette prices and how excise differentials translated into differences in tax burden and affordability. Finally, it estimates the pass-through of HTP and cigarette excise changes into HTP retail prices and assesses whether the broader pricing pattern may be interpreted as consistent with strategic industry pricing under the favorable tax treatment of HTPs.

## METHODS

### Study design and setting

A secondary data analysis was conducted using administrative and publicly available market data from Serbia. Monthly price and excise series covered Q3 2018, when HTPs entered the Serbian market, through Q1 2025, while aggregate tobacco sales data covered 2018–2023. In the panel models, the unit of observation was the brand-month, with 153 observations across two representative HTP brand series.

### Data sources

Several official data sources were combined to analyze HTP prices, taxation, and market developments in Serbia. Monthly data on HTP and cigarette prices and excise taxes were collected from official gazettes. Aggregate tobacco sales volumes and values were obtained from the Tobacco Administration for 2018–2023. Consumer prices and wages were taken from the Statistical Office of the Republic of Serbia (SORS), while materials from the Ministry of Finance were used to interpret the legal framework and, where necessary, reconstruct the tax series.

Because Serbia does not publish brand-level monthly data on retail prices per pack and sales volumes for HTPs, we could not directly observe monthly HTP prices per pack or calculate monthly weighted average retail prices for the HTP market. This required additional reconstruction of HTP price and tax variables from official administrative sources, as described in Supplementary file Box S1.

### Construction of price, sales, and tax variables

*HTP price series*

Official gazettes report HTP prices per kilogram of tobacco rather than per pack. Pack-level HTP prices were therefore reconstructed from the announced kilogram prices (Supplementary file Box S1). Throughout the analysis, an HTP pack refers to a 20-stick pack of HTP tobacco consumables. This reconstruction involved two complications. First, new HTP brands and variants often entered the market with different tobacco content per pack. Second, replacement of older products was gradual, and overlapping sales periods could not be identified precisely because brand-level sales data were unavailable. Brand entry timelines and changes in pack tobacco content are shown in Supplementary file Figures S1a and S1b.

Because monthly sales by brand were unavailable, representative brand prices were used as proxies for market price movements. For Philip Morris International (PMI), Heets and Terea, both HTP tobacco-stick brands, were combined into a single representative series because Terea replaced Heets in 2023 and entered the market at the same pack price. For British American Tobacco (BAT), Neo, another HTP tobacco-stick brand, was used because it had a continuous market presence and relatively stable pricing despite changes in tobacco weight across product revisions. Within a given HTP brand, price differences across variants were rare, which supports this approximation. At the same time, these representative series should be interpreted as approximations of broader market price movements rather than exact market averages.

*Sales, packs, affordability, and tax burden*

To describe market expansion, aggregate sales quantities and values reported by the Tobacco Administration were used. HTP and roll-your-own tobacco quantities were converted into estimated pack equivalents. This allowed HTP, manufactured cigarette, and roll-your-own sales to be expressed using a common pack-based metric. These estimates depend on assumptions about tobacco content and should therefore be interpreted as approximate indicators of market structure rather than exact sales measures.

To examine affordability, HTP prices were expressed in nominal and real terms and considered alongside wage developments. Nominal prices refer to observed current prices. Real prices were derived using the consumer price index from SORS, with 2006 as the base year. Affordability was assessed primarily through changes in real HTP prices and real wage growth over time rather than through a standard relative income price (RIP) measure. This approach captures changes over time within Serbia but limits direct comparability with studies using RIP-based affordability indicators.

In Serbia, HTPs are taxed per kilogram of tobacco, while cigarettes are taxed per pack. To analyze tax differentials between HTPs and cigarettes, we converted the HTP excise burden from a per-kilogram tobacco basis to a per-pack equivalent. The taxation of HTPs was jointly determined by decisions on minimum excise for manufactured cigarettes and by the provisions of the Law on Excise, which specify the proportion of minimum cigarette excise applied to HTPs^15^. HTP excise is therefore legally linked to the minimum excise on cigarettes through a phased schedule. The full legislative schedule and the corresponding reconstructed excise series are presented in Supplementary file Table S1, while the conversion of HTP excise from kilogram to pack level is illustrated in Supplementary file Box S2. We converted HTP excise per kilogram into excise per pack using the estimated tobacco content per pack shown in Supplementary file Figures S1a and S1b. For descriptive comparisons, we decomposed retail prices into excise, value-added tax (VAT), and the residual net-of-tax component. Because Serbian retail prices are VAT-inclusive and the standard VAT rate is 20%, the VAT component was calculated as (retail price×20)/120^16^. The net-of-tax component was then calculated as retail price minus VAT and excise, while the excise burden was calculated as the share of excise in the final retail price.

### Statistical analysis

Descriptive statistics were used to summarize developments in HTP sales, prices, affordability, and taxation. The pass-through of excise taxes into HTP retail prices was then estimated using monthly panel data for Q3 2018 – Q1 2025, following recent empirical work on HTP taxation and pricing. The baseline model relates monthly changes in HTP retail prices to monthly changes in HTP excise per pack, with consumer prices and wage growth included as macroeconomic controls. In this specification, the dependent variable is the change in HTP retail price, while the main independent variable is the change in HTP excise per pack. The models were estimated in first differences to remove time-invariant brand fixed effects and help address potential non-stationarity in the price and tax series. A coefficient below 1 indicates incomplete pass-through, whereas a coefficient above 1 indicates over-shifting. Two alternative HTP-price specifications were also estimated: one including only cigarette excise changes and one including both HTP and cigarette excise changes. These models were used to examine both direct pass-through from HTP taxation and indirect associations between cigarette taxation and HTP prices. Because the models estimate contemporaneous associations, they do not capture anticipatory or delayed price responses.

To assess whether the results were sensitive to joint pricing across product categories, a seemingly unrelated regression (SUR) system was also estimated for HTP and cigarette prices. The SUR system allowed the two price equations to be estimated jointly while accounting for contemporaneous correlation in unobserved pricing shocks across product categories. It was used to assess whether joint estimation changed the HTP-price results, while substantive interpretation focused on the HTP equation. Finally, a gap model was estimated relating the difference between cigarette and HTP prices to the difference between their respective excise taxes. In all models, tax variables refer to excise only, without VAT.

## RESULTS

### HTP market developments in Serbia

*Sales and market expansion*

We found a continuous expansion of the HTP market in Serbia after its entry in 2018. Based on Tobacco Administration data, both the quantity of HTP tobacco sold and the value of HTP sales increased over time (Figure 1). Growth was relatively modest in 2019 and 2020, but accelerated markedly from 2021 onward. By 2023, the quantity of HTP tobacco sold was approximately 2.5 times higher than in 2020 (for annual growth rates, see Supplementary file Figure S2).

**Figure 1:**
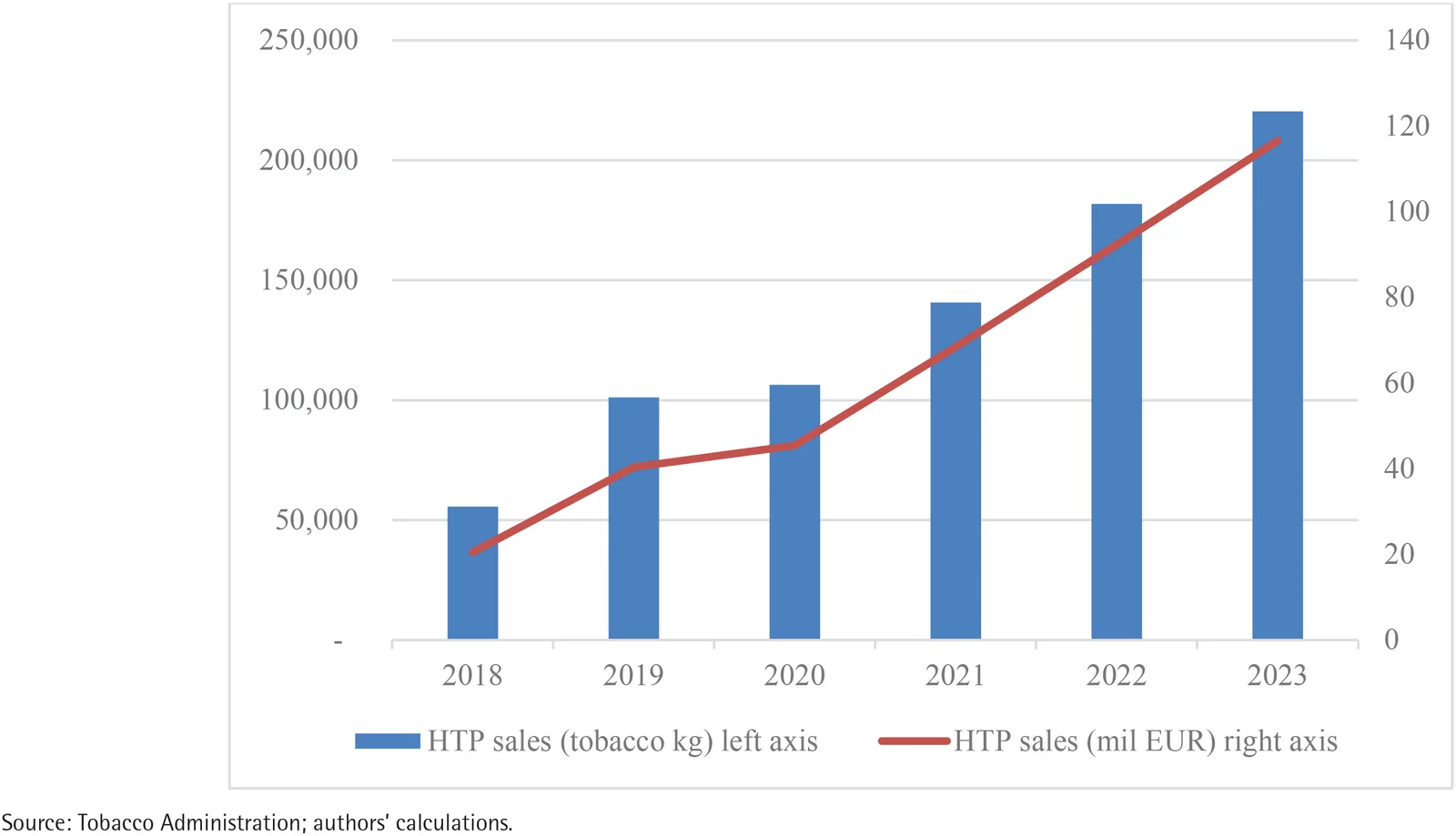
Annual quantity and sales value of heated tobacco products in Serbia based on administrative data, 2018–2023

The increase was visible not only in absolute terms, but also relative to other tobacco products. After converting HTP tobacco sales into estimated numbers of packs, we found that HTPs accounted for around 2.5% of all tobacco packs sold in 2019. By 2023, this share had increased to 6.25% (Figure 2). This indicates that HTPs moved from a marginal product category to a more visible component of the Serbian tobacco market within a relatively short period.

**Figure 2:**
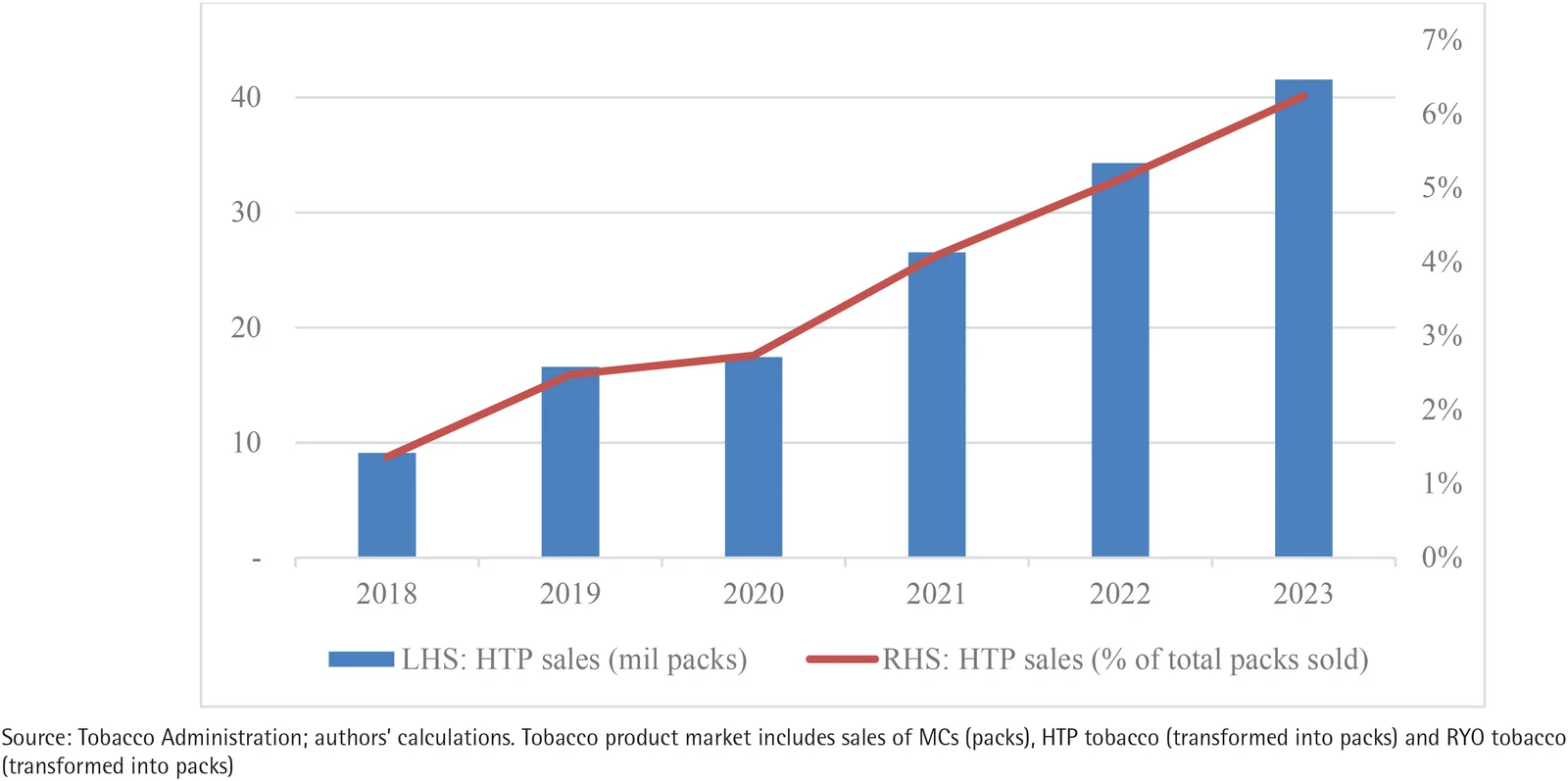
Estimated annual HTP pack-equivalent sales and share of total tobacco packs sold in Serbia based on administrative data, 2018–2023

*Relative price dynamics and affordability*

HTP prices increased in nominal terms throughout the observed period. The annual average price of the highest selling HTP brand rose from RSD 270 in 2018 to RSD 367 in Q1 2025. Expressed in euro, the price increased from EUR 2.28 to EUR 3.13 over the same period. However, the real price trend was less linear. Real prices increased until 2021, but then declined, so that in 2023 and 2024 HTP prices were lower in real terms than in 2018. At the same time, real wages continued to grow, which implies a substantial increase in affordability (Table 1).

**Table 1:** Average annual nominal and real prices of the highest-selling HTP brand (PMI Heets/Terea) per pack in Serbia, Q3 2018 – Q1 2025

*Year*	*RSD*	*RSD real (2006 = 100)*	*EUR*	*EUR real (2006 = 100)*	*Real wage growth (%)*
**2018**	270	138	2.28	1.90	
**2019**	283	143	2.40	1.97	8.37
**2020**	299	149	2.54	2.07	7.68
**2021**	320	153	2.72	2.16	5.30
**2022**	329	141	2.80	2.03	1.67
**2023**	339	129	2.89	1.98	2.10
**2024**	356	129	3.04	2.03	9.03
**2025**	367	130	3.13	2.04	4.87

Source: Authors’ calculations. Q1: first quarter of year. RSD: 1000 Serbian Dinars about US$9.9.

Relative price comparisons with cigarettes showed a clear repositioning of HTPs over time. At market entry, the representative HTP price was located between the prices of medium and premium cigarette brands. Later, HTP prices increased more slowly than cigarette prices. As a result, the price gap between HTPs and premium cigarettes widened, while the gap between HTPs and the representative medium-priced cigarette brand narrowed substantially. By the later years of the series, HTP prices had moved close to the medium cigarette segment (Figure 3).

**Figure 3:**
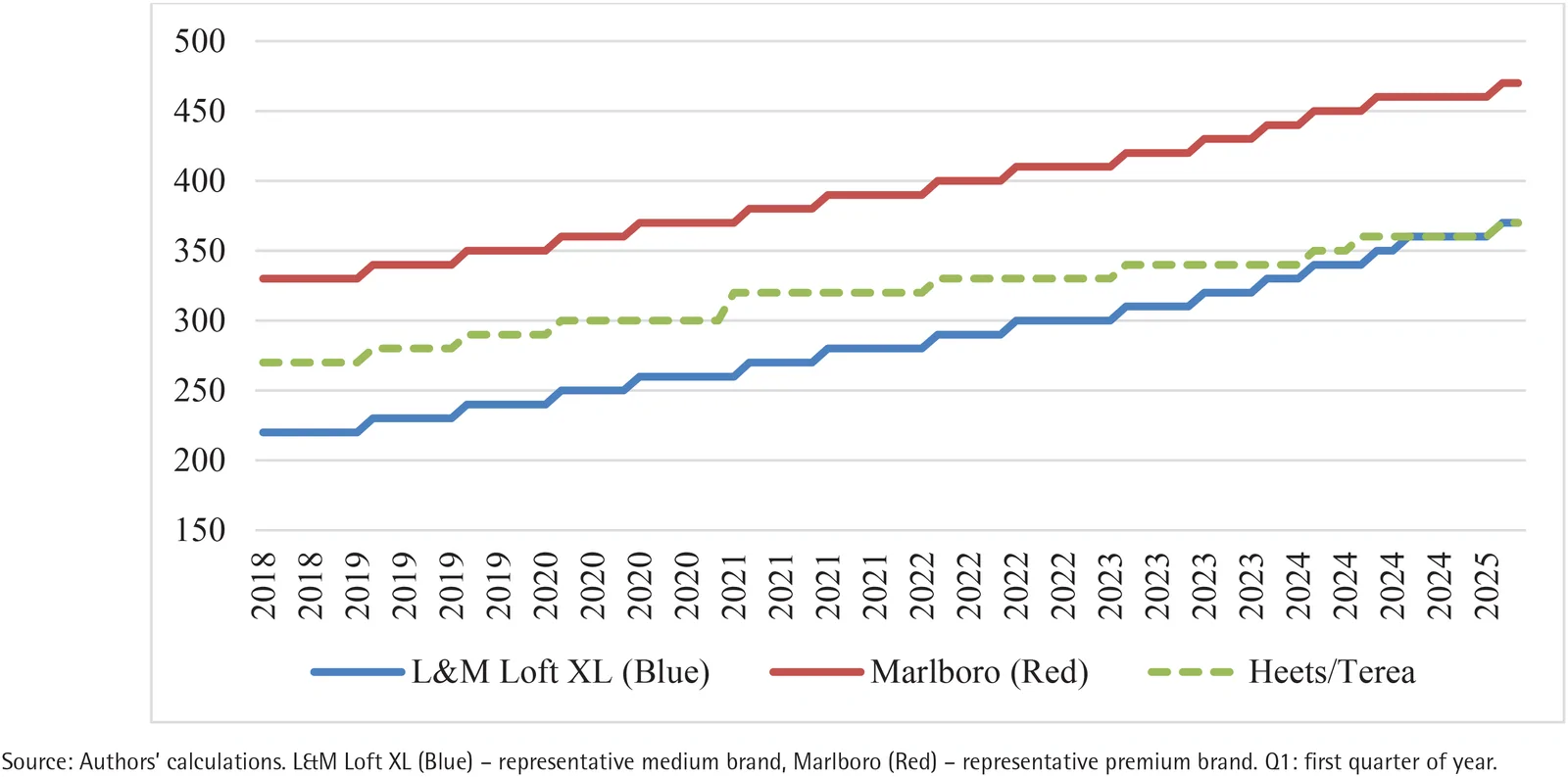
Monthly retail prices of representative HTP, medium-priced cigarette, and premium cigarette brands in Serbia, Q3 2018 – Q1 2025

Annual comparisons of HTP and cigarette weighted average retail price (WARP) point in the same direction: HTPs were initially slightly more expensive than the overall cigarette average, but from 2021 onward cigarette WARP increased faster than HTP WARP, making HTPs relatively more affordable than cigarettes on average (Supplementary file Figure S3).

*Excise structure and tax burden*

The Serbian excise system generated a persistent tax advantage for HTPs relative to manufactured cigarettes. Although HTP excise increased substantially during the observed period, the excise burden per HTP pack remained much lower than for cigarettes. The reconstructed mid-year price decomposition of the highest-selling HTP brand shows that excise tax per pack increased from EUR 0.15 in 2018 to EUR 0.41 in 2024. Over the same period, the excise share in the final retail price rose from 6.66% to 13.39%, while the total tax share, calculated after extracting VAT from the tax-inclusive price, increased from 23.33% to 30.06% (Table 2).

**Table 2:** Mid-year decomposition of the nominal retail price of the highest-selling 20-stick HTP brand pack (PMI Heets/Terea) in Serbia, 2018–2024

	*Mid 2018*	*Mid 2019*	*Mid 2020*	*Mid 2021*	*Mid 2022*	*Mid 2023*	*Mid 2024*
**Price** (EUR)	2.29	2.38	2.55	2.72	2.81	2.90	3.08
**Excise tax** (EUR)	0.15	0.16	0.17	0.23	0.28	0.32	0.41
**VAT** (EUR)	0.38	0.40	0.43	0.45	0.47	0.48	0.51
**Net-of-tax price** (EUR)	1.76	1.82	1.95	2.04	2.06	2.10	2.16
**Excise tax** (%)	6.66	6.76	6.68	8.62	9.84	10.91	13.39
**Total tax** (%)	23.33	23.43	23.35	25.29	26.51	27.58	30.06

Source: Authors’ calculations. VAT is extracted from the VAT-inclusive retail price as (price×20)/120. Total tax equals excise plus VAT.

Despite this increase, HTPs remained lightly taxed in comparison with cigarettes. In 2024, excise on a 20-stick HTP pack amounted to EUR 0.41, which was less than half of the specific excise on cigarettes, reported at EUR 0.85. The relatively low tax burden translated into a large net-of-tax component of the HTP retail price. The net-of-tax price of the leading HTP brand rose from EUR 1.76 in 2018 to EUR 2.16 in 2024. These descriptive results show that the increase in HTP taxation was not matched by a comparable increase in the tax share of the final retail price.

To assess the commercial implications of this price structure, we estimated that the aggregate net-oft-ax value of HTP sales increased from approximately EUR 16.7 million in 2018 to EUR 91.4 million in 2023. HTPs accounted for 6.82% of the combined net-of-tax sales value of HTPs and the representative medium-priced cigarette brand in 2018 and 18.83% in 2023, while their corresponding shares of combined pack sales were only 1.38% and 6.51%. In 2023, the net-of-tax value per HTP pack was EUR 2.20, more than three times the EUR 0.66 estimated for the representative medium-priced cigarette pack. This disproportion shows that the observed market shift toward HTPs has directed an increasing share of aggregate net-of-tax sales value to the HTP segment and, under the existing tax structure, creates a clear commercial incentive for further expansion. These estimates refer to net-of-tax sales value rather than accounting profit and depend on representative prices and pack conversions (Table 3).

**Table 3:** Estimated annual net-of-tax sales value of HTPs and a representative medium-priced cigarette brand in Serbia based on administrative quantities and representative prices, 2018–2023

*Year*	*HTP packs*	*Cigarette packs*	*HTP NoT per pack*	*Cigarette mid NoT per pack*	*HTP NoT Total*	*Cigarette mid NoT Total*	*HTP NoT share (%)*	*HTP share (%)*
**2018**	9125533	651516536	1.83	0.35	16699725	228030788	6.82	1.38
**2019**	16595233	643979869	1.90	0.37	31530943	238272552	11.69	2.51
**2020**	17434677	607454789	2.05	0.47	35741088	285503751	11.13	2.79
**2021**	26543949	606277764	2.12	0.53	56273172	321327215	14.90	4.19
**2022**	34320915	613466882	2.17	0.59	74476386	361945460	17.07	5.30
**2023**	41568017	597354350	2.20	0.66	91449637	394253871	18.83	6.51

Source: Authors’ calculations based on Tobacco Administration data and official gazettes. NoT: net-of-tax component of retail price, in EUR. Cigarette mid: representative medium-priced cigarette brand. HTP NoT share is the share of HTP aggregate net-of-tax value in the combined HTP and cigarette net-of-tax total. HTP share is the share of HTP packs in combined HTP and cigarette packs sold. These values are not measures of accounting profit.

### Pass-through effects

Table 4 shows the results of the pass-through models. In the baseline specification (Model 1), the coefficient on the HTP excise tax increment was 0.2588 (SE=0.1677; 95% CI: -0.0699–0.5875). The coefficient was positive and below one, but the confidence interval included zero. The point estimate therefore does not support full pass-through, but it also does not provide clear evidence of a positive direct association between HTP excise changes and HTP retail prices.

**Table 4:** First-difference panel regression estimates of HTP retail price changes in Serbia, Q3 2018 – Q1 2025 (N=153 brand-month observations)

*Variables*	*HTP price increment (RSD)*				*Price gap increment (RSD)*
	*Model 1*	*Model 2*	*Model 3*	*Model 4*	*Model 5*
**HTP tax increment (RSD)**	0.2588 (0.1677) [-0.0699–0.5875]		0.0894 (0.1597) [-0.2236–0.4024]	0.0853 (0.1576) [-0.2236–0.3942]	
**MC tax increment (RSD)**		0.9856*** (0.1898) [0.6136–1.3576]	0.9622*** (0.1948) [0.5804–1.3440]	0.9623*** (0.1924) [0.5852–1.3394]	
**Tax gap increment (RSD)**					0.7287*** (0.1402) [0.4539–1.0035]
**R^2^ (%)**	1.7	15.3	15.5	15.4	15.4

Source: Authors’ calculations. Tax refers only to excise (VAT not included). Model 1: baseline. Models 2–5 use a representative medium-segment cigarette series. Model 4 is the HTP-price equation from the SUR system, which allows contemporaneous correlation across HTP and cigarette pricing equations. Gaps are calculated as cigarette minus HTP values. Standard errors are in parentheses and 95% confidence intervals in square brackets.

*** p<0.001.

When we related HTP price changes to cigarette excise changes, the estimated association was much stronger. In Model 2, the coefficient on the cigarette tax increment was 0.9856 (SE=0.1898; 95% CI: 0.6136–1.3576). The estimate remained very similar when HTP and cigarette excises were entered jointly in Model 3, where the cigarette tax coefficient was 0.9622 (SE=0.1948; 95% CI: 0.5804–1.3440), while the HTP excise coefficient was 0.0894 (SE=0.1597; 95% CI: -0.2236–0.4024). The corresponding HTP price equation from the SUR system (Model 4) produced almost identical results, with a cigarette tax coefficient of 0.9623 (SE=0.1924; 95% CI: 0.5852–1.3394) and an HTP excise coefficient of 0.0853 (SE=0.1576; 95% CI: -0.2236–0.3942). Taken together, these models indicate that changes in HTP prices were much more closely aligned with cigarette excise changes than with HTP excise changes. The similarity between Model 3 and Model 4 also indicates that joint estimation did not materially alter the HTP-price results.

The relative-price model (Model 5) also pointed in the same direction. The coefficient on the monetary tax gap between cigarettes and HTPs was 0.7287 (SE=0.1402; 95% CI: 0.4539–1.0035), indicating that a 1 RSD increase in the cigarette–HTP excise gap was associated with an approximately 0.73 RSD increase in the corresponding retail-price gap.

The explanatory power of the HTP price models was modest. R^2^ was 1.7% in Model 1 and around 15% in Models 2–5. Inflation and wage growth were included as controls, but neither variable was statistically significant and their inclusion did not materially improve model fit. Overall, the regression results show that HTP excise increases were not clearly reflected in HTP retail prices, whereas cigarette excise changes were strongly associated with HTP price dynamics. These estimates describe associations in price changes rather than a complete or causal model of HTP pricing.

## DISCUSSION

Our findings show that the expansion of HTPs in Serbia took place under a tax structure that was substantially more favorable than the one applied to manufactured cigarettes. During the observed period, HTP sales increased steadily, their market share became more visible, and their prices moved closer to the medium cigarette segment, while the tax burden on HTPs remained much lower than on cigarettes even after successive excise increases. Taken together, these results suggest that the Serbian excise system created conditions in which HTPs could become more competitive without facing a comparable fiscal burden. The growing share of aggregate net-of-tax sales value indicates that HTPs became an increasingly important revenue component for the industry. Because HTPs generated a far larger share of net-of-tax sales value than of pack sales, the current tax structure creates a clear commercial incentive to expand this segment and, if left unchanged, is likely to reinforce the industry’s strategic shift from manufactured cigarettes toward HTPs.

The price results are important for two reasons. First, nominal HTP prices increased over time, but real prices did not follow the same pattern. After 2021, HTPs became cheaper in real terms than at market entry, while real wages continued to grow, implying greater affordability during a period of market expansion. Second, HTP prices gradually converged toward the medium cigarette segment rather than remaining closer to premium cigarettes. Although we cannot directly observe consumer switching at the individual level, this pricing pattern is consistent with the idea that HTPs became increasingly accessible to smokers outside the premium segment and with substitution incentives documented in the HTP demand literature^9,10^. In that sense, relative price positioning appears to be at least as important as absolute price growth.

Our pass-through estimates add a more nuanced part of the story. The direct association between HTP excise changes and HTP retail prices was positive and below one, but its confidence interval included zero. Although the same interval excluded full pass-through, the low explanatory power of the baseline model limits the substantive interpretation of the estimate. We therefore cannot draw a firm conclusion about the magnitude of direct HTP pass-through and do not interpret this result as strong standalone evidence of systematic under-shifting. However, the broader pattern across models remains informative. HTP price changes were much more closely aligned with cigarette excise changes than with changes in HTP excise itself, and larger tax gaps between cigarettes and HTPs were associated with larger price gaps between the two product categories. This suggests that HTP pricing in Serbia was shaped not only by the tax treatment of HTPs themselves, but also by their position within the wider tobacco price structure. In practical terms, this broader pattern may be interpreted as consistent with strategic relative-price positioning with reference to cigarettes, but it does not establish industry intent or a causal pricing mechanism.

These findings are broadly consistent with previous work on HTP taxation and pricing. Earlier cross-country studies reported that cigarette excise increases are often fully shifted, or even over-shifted, while HTP excise increases are more frequently under-shifted^7^. The Serbian case does not provide a clean and precise estimate of direct HTP under-shifting, but it points in the same general direction. It also resembles the pattern reported for Montenegro, where large HTP tax increases were followed by much smaller increases in HTP retail prices^11,12^. Recent evidence from Ukraine provides an additional comparator by showing price smoothing and differentiated timing of adjustments around scheduled reforms^13^. Our results therefore fit the broader literature suggesting that tax design and the timing of tax changes can shape relative prices across product categories.

From the policy perspective, the main implication is that closer alignment of HTP and cigarette taxation may be considered as a policy option. The WHO FCTC recognizes HTPs as tobacco products subject to the Convention’s regulatory measures^17^, and Article 6 guidance emphasizes effective tax and price measures as part of comprehensive tobacco control^18^. In this context, policymakers could consider a unit-based specific excise and an ad valorem component to reduce tax differentials and opportunities for strategic product positioning. However, our econometric analysis did not directly evaluate the effects of these alternatives on consumption, substitution, public revenue, or public health.

A third implication concerns fiscal transparency and market monitoring. The Serbian case showed that minimum excise values were not always published or updated in a transparent manner, especially during part of the 2021–2024 period (for detailed chronology see Supplementary file Table S1). In a rapidly changing market, such opacity weakens fiscal governance and makes independent evaluation more difficult. Better monitoring of retail prices, product characteristics, promotions, and brand-level sales would improve both tax administration and policy evaluation, and would allow market-weighted pass-through analysis.

### Limitations

This study has several limitations. We reconstructed monthly HTP pack prices from kilogram-based prices published in official gazettes rather than directly observed brand-level retail data. In addition, the absence of monthly sales volumes by brand prevented us from calculating weighted average HTP prices and required us to use representative brand series. Although the panel contained 153 brand-month observations, identifying variation came from a limited number of tax and price changes across only two representative HTP series. Promotions, product replacement, brand repositioning, device launches, and other regulatory or market changes may have affected prices. Scheduled excise reforms may also allow anticipatory or delayed price adjustments that may not be captured by the contemporaneous first-difference models. Inflation and wage growth were included as controls, but other time-varying factors could not be fully isolated. Affordability was assessed using real prices and real wages rather than a standard RIP measure, which limits comparability with RIP-based studies. These limitations, together with the single-country observational design and modest model fit, require caution when interpreting the pass-through estimates and preclude causal attribution of price changes solely to excise adjustments.

This study also has several strengths. We reconstructed a continuous HTP price and tax series from market entry onward and integrated it with administrative sales data and explicit pass-through models. The results consistently show a persistent tax differential, a growing relative price advantage of HTPs over cigarettes, and HTP price movements closely connected to the wider cigarette price structure. These patterns are policy relevant, but the size and timing of direct tax pass-through remain uncertain.

## CONCLUSIONS

Our study showed that sales of HTPs expanded rapidly in Serbia between 2018 and 2025 while remaining subject to a substantially lower effective tax burden than cigarettes. During this period, their real prices declined after 2021, their market positioning moved toward the medium cigarette segment, and their contribution to aggregate net-of-tax sales value increased. The direct HTP excise coefficient was below full pass-through, but its confidence interval included zero. By contrast, cigarette excise changes were more strongly associated with HTP price changes. Taken together, these findings support a cautious interpretation that HTP pricing was connected to relative positioning within the broader tobacco market.

From a policy perspective, closer alignment of HTP and cigarette taxation may be considered as an option consistent with WHO FCTC treatment of HTPs as tobacco products and its guidance on tax and price measures^17,18^. Policymakers could consider replacing the weight-based HTP excise with a specific excise per 1000 sticks and introducing an ad valorem component aligned with cigarettes. We did not directly evaluate the effects of these alternatives, and future research should assess their effects on prices, consumption, substitution, public revenue, and public health.

## Supplementary Material



## Data Availability

The data supporting this research are available from the authors on reasonable request.
