# Supplementary material for: Heated tobacco products in Serbia: Tax differentials, relative prices, and excise tax pass-through
Source: Tob Induc Dis. 2026 Oct 5;24:10.18332/tid/231126. doi: 10.18332/tid/231126 (PMC13640022; doi:10.18332/tid/231126)
Supplement: Supplementary file 1 [file TID-24-184-s1.pdf]

Tobacco Induced Diseases

Supplementary file

© 2026 Zdravković A. et al.

DOI:
10.18332/tid/231126

The content has been provided by the author(s) and has not been reviewed, verified, or endorsed by European Publishing. It may not have undergone peer review. The views, opinions, and recommendations expressed are solely those of the author(s) and do not necessarily reflect the position of European Publishing. European Publishing accepts no responsibility or liability for any consequences arising from the use of, or reliance on, this content.

SUPPLEMENTARY MATERIAL

Box S1: Constructing time series of the HTP prices

In the absence of data on HTP prices per pack, the prices per HTP tobacco kg are transformed into prices per pack in the following way. We started from the current price of HTP per pack observed in cigarettes stores (column Price per HTP pack in RSD in Table S1) and then computed number of packs per kg of HTP tobacco as price per kg / price per pack. For instance, price per tobacco kg of Terea in 2025 Q1 was RSD 69,811.32 and price per pack was RSD 370, so that number of packs per tobacco kg is calculated as 69,811.32/370 = 188.68. Similarly, we compute current quantity of tobacco per pack as 1,000g / number of packs. In particular case of Terea, dividing 1,000 by 188.68 gives exactly 5.3g per pack.

Bearing in mind the practice of industry in Serbia to increase prices of cigarettes and HTPs for exactly 10 RSD no matter how much excise increases, we recursively compute prices per pack whenever change in price per HTP tobacco kg is announced in official gazette and then check consistency in terms of number of packs per kg/grams per pack.

Subsequently, monthly time series of prices is generated in line with previous calculations and changes of HTP tobacco kg prices announced in the official gazettes. For instance, if the official gazette dated 7 September 2023 announced that new price of Terea tobacco kg is RSD 64,150.94, we computed price per pack of Terea (RSD 340 in this case) and set this price for the September 2023 – January 2024 period, as the new price of Terea tobacco kg is announced on 6 February 2024.

Figure S1a: Timeline of PMI HTPs introduction in Serbia

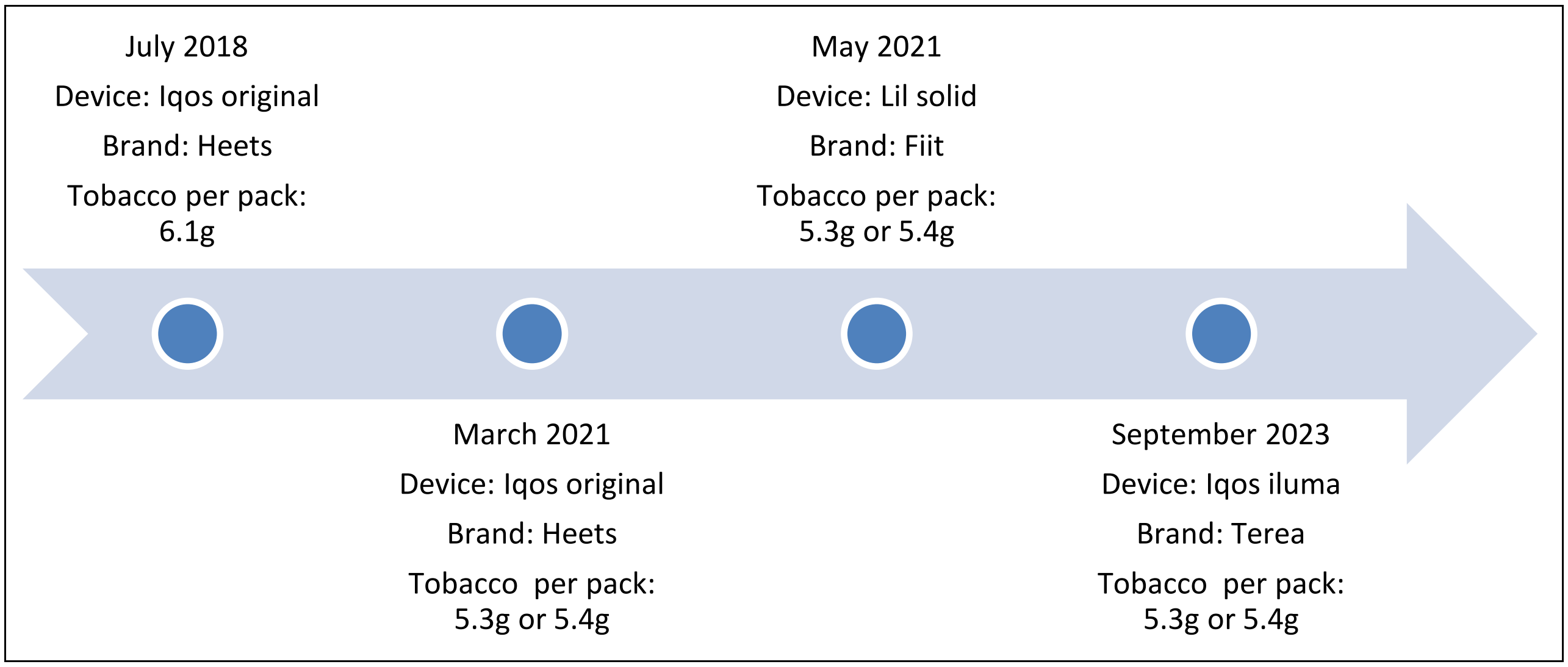

Source: Official gazettes, authors' calculation; Note: timeline refers to dates when the given brand appeared for the first time in official gazette or when the change in quantity of tobacco occurred

Figure S1b: Timeline of BAT HTPs introduction in Serbia

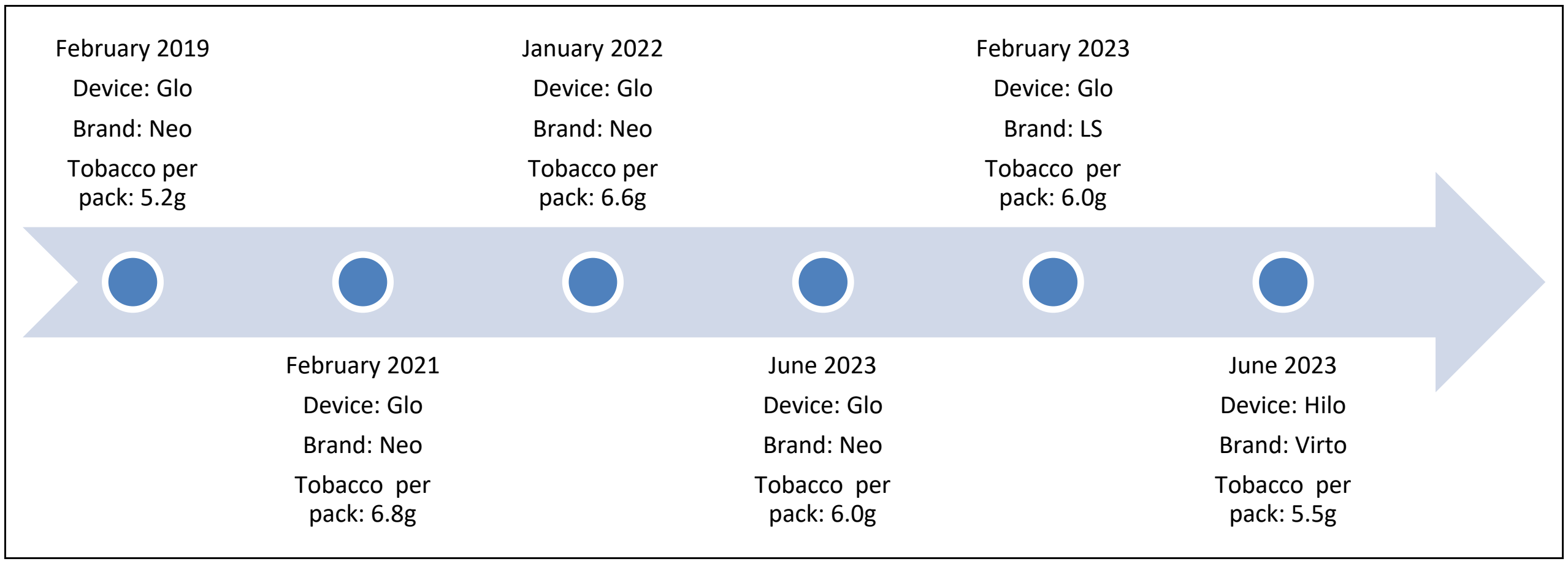

Source: Official gazettes, authors' calculation; Note: timeline refers to dates when the given brand appeared for the first time in official gazette or when the change in quantity of tobacco occurred. LS – Lucky Strike.

Table S1: Excise on HTPs and minimum excise on cigarettes

Legislation	Came into force	WARP	Min exc per 20-stick pack (20 g of tob)	Min exc per 1000 sticks (1 kg of tob)	Exc per HTP tob kg
OG 52/18	07.07.2018	241.42	147.42	7,371.00	2,948.40
OG 84/18	03.11.2018		148.86	7,443.00	2,977.20
OG 12/19	23.02.2019	256.16	155.23	7,761.50	3,104.60
OG 51/19	20.07.2019		156.75	7,837.50	3,135.00
OG 91/20	27.06.2020	274.24	164.23	8,211.50	3,284.60
OG 104/20	01.08.2020		165.75	8,287.50	3,315.00
LoE – 60%	01.01.2021		165.75	8,287.50	4,972.50
OG 15/21	20.02.2021	293.14	173.49	8,674.50	5,204.70
OG 74/21	24.07.2021		174.99	8,749.50	5,249.70
LoE – 70%	01.01.2022		174.99	8,749.50	6,124.65
LoE – 80%	01.01.2023		174.99	8,749.50	6,999.60
OG 75/23	01.10.2023		202.13	10,106.50	8,085.20
LoE – 90%	01.01.2024		202.13	10,106.50	9,095.85
OG 94/24	06.12.2024		218.34	10,917.00	9,825.30
LoE – 100%	01.01.2025		218.34	10,917.00	10,917.00
OG 13/25	15.02.2025	393.05	233.36	11,668.00	11,668.00

Note: Official gazettes (OG) in which decisions on minimum excise were announced. Minimum excise values on 01.01.2022 and 01.01.2023 are imputed based on Custom Administration documents. WARP refers to the weighted average retail price of MCs in the previous year. LoE refers to the changes automatically imposed by the Law on Excise

Box S2: Computing excise per HTP pack in Serbia

As previously mentioned, HTPs in Serbia are taxed per kg of tobacco. Therefore, computing excise on HTP pack requires additional computational steps.

In the first step, HTP excise per kg is computed based on minimum excise,

HTP excise (kg) = Minimum excise (cigarette pack) * 50 (number of cigarette packs in kg) * HTP excise/min excise,

where HTP excise/min excise refers to the % of minimum excise on cigarettes applied to HTP as stipulated by the LoE.

In the second step, HTP excise per pack is obtained by computing HTP excise per gram of HTP tobacco and then multiplying this amount by quantity of tobacco in HTP pack,

HTP excise (pack) = HTP excise (kg) / 1000 (g) * HTP tobacco weight (g/pack),

For instance, minimum excise in mid-2023 was RSD 174.99 and respective HTP excise/min excise ratio was 80% (see Supplementary Table S1), while quantity of tobacco in Terea pack was 5.3 g. The calculation of excise on Terea pack in this case is summarized as follows:

Minimum excise	RSD 174.99 per pack
HTP excise/min excise	0.8 (80%)
HTP tobacco weight	5.3 g/pack
HTP excise (kg)	RSD 174.99 per pack * 50 packs in kg * 0.8 = RSD 6,999.60 per kg
HTP excise (pack)	RSD 6,999.60 per kg / 1,000 g * 5.3g per pack = RSD 37.10 per pack

Figure S2: Y-o-Y growth rates of HTP tobacco sales

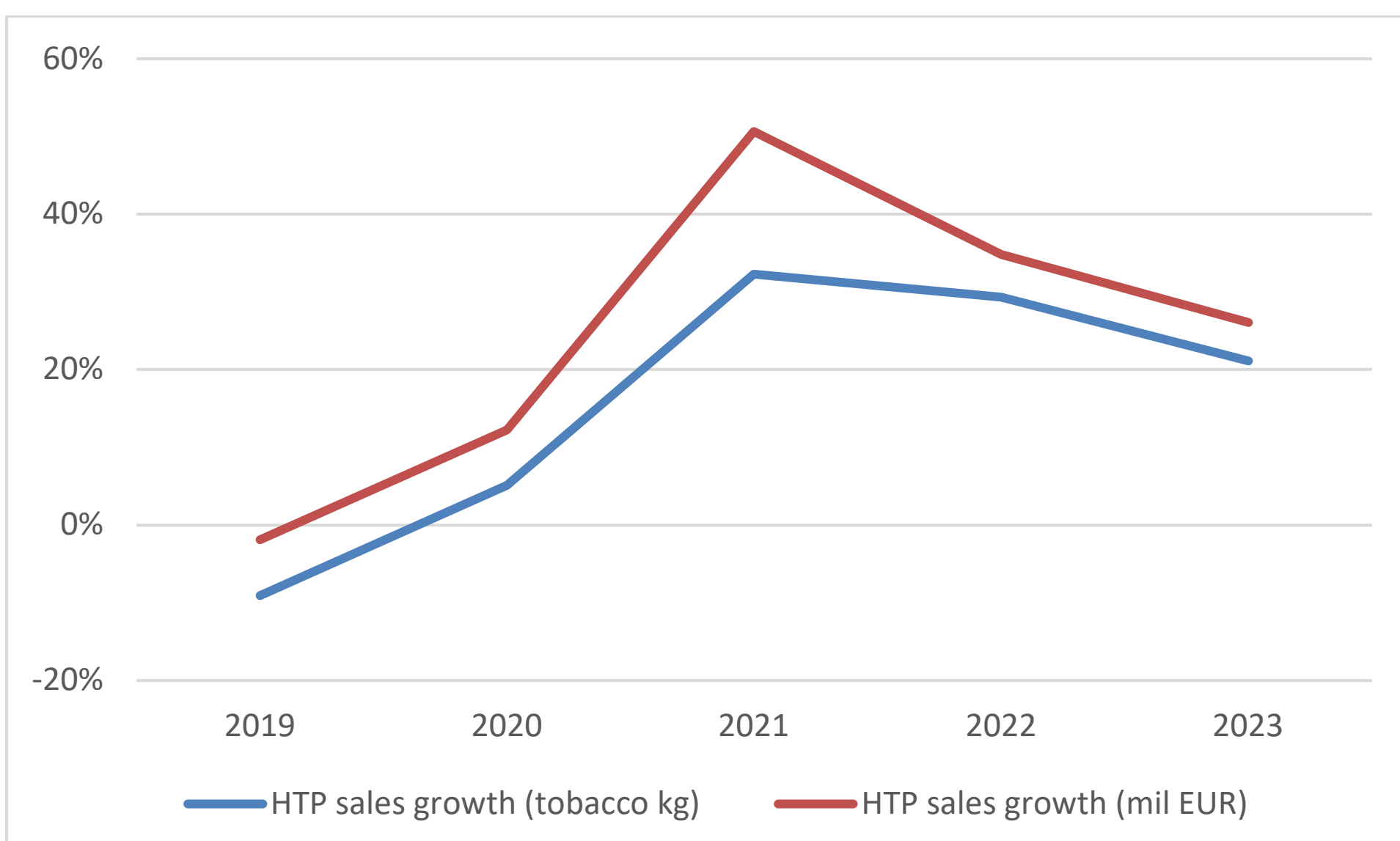

Source: Tobacco Administration, authors' calculation; Note: rate for 2019 is annualized

Figure S3: Cigarette WARP vs. HTP WARP, annually in RSD

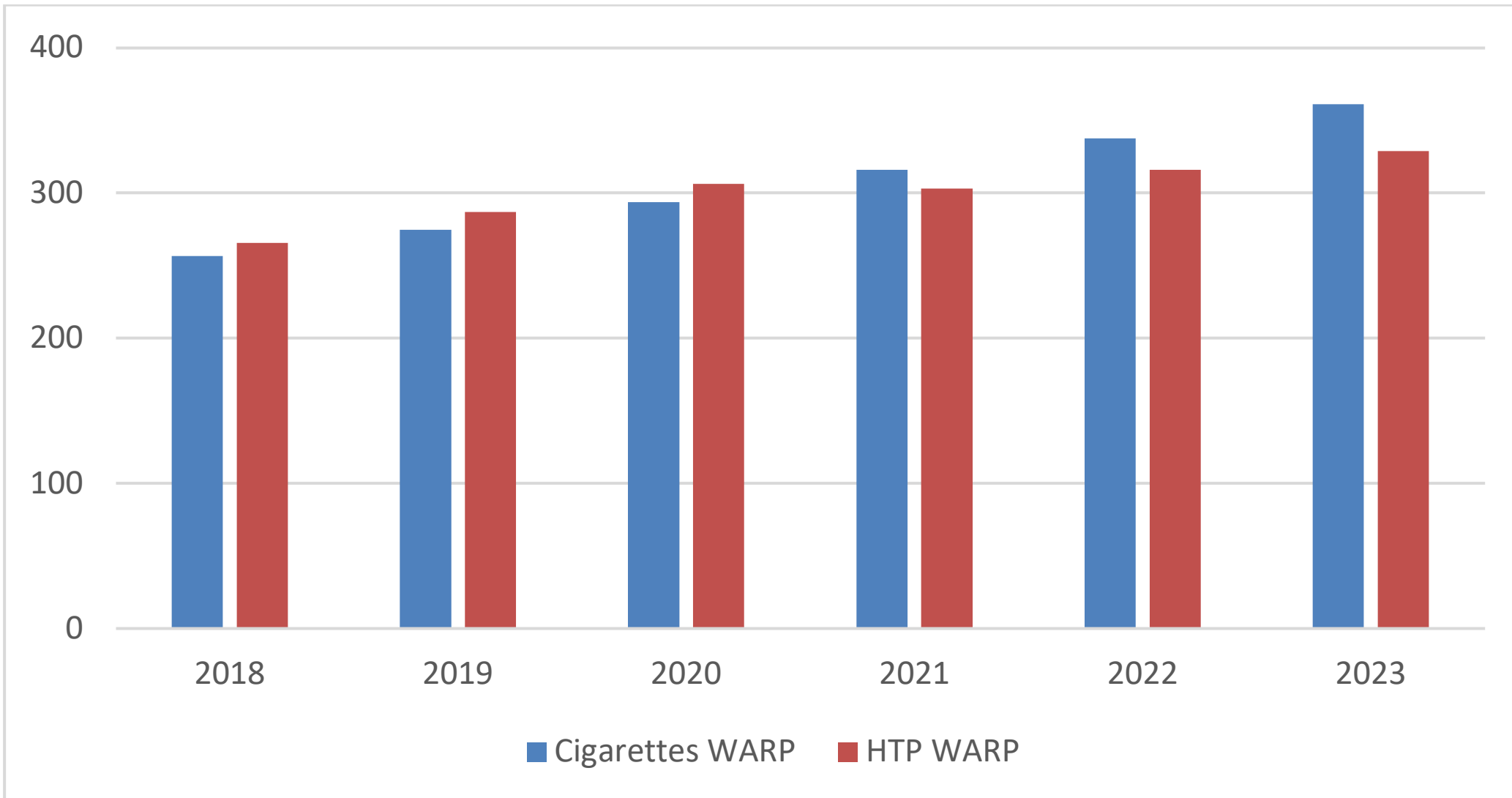

Source: Tobacco Administration, authors' calculation
